# Supplementary material for: The Calcium Channel Subunit Gamma-4 as a Novel Regulator of MafA in Pancreatic Beta-Cell Controls Glucose Homeostasis
Source: Biomedicines. 2022 Mar 25;10(4):770. doi: 10.3390/biomedicines10040770 (PMC9030882; doi:10.3390/biomedicines10040770)
Supplement: Supplementary file 1 [file biomedicines-10-00770-s001.zip › biomedicines-1630825-supplementary.pdf]

Supplementary Table S1

Supplementary Table S1. Information list of human islet donors used for experiment.

	Age	Gender	BMI (kg m^{-2})	HbA1c (%)	Diabetes status
Donor 1	50	Male	22.7	5.4	Non diabetic
Donor 2	39	Male	24.7	5.8	Non diabetic
Donor 3	67	Male	32.5	5.8	Non diabetic
Donor 4	64	Female	26.9	5.7	Non diabetic
Donor 5	57	Male	30.4	NA	Non diabetic
Donor 6	56	Male	24.3	5.7	Non diabetic
